# Physical Activity in the Management of Diabetic Neuropathic Pain: A Narrative Review of Mechanisms, Evidence, and Clinical Perspectives

**DOI:** 10.7759/cureus.94960

**Published:** 2025-10-20

**Authors:** Jaouher Dhouibi, Houda Migaou, Amr Chaabeni, Amine Kalai, Sana Salah, Soumaya Boudokhane, Zohra Ben Salah Frih, Anis Jellad

**Affiliations:** 1 Physical Medicine and Rehabilitation, University of Monastir, Faculty of Medicine, Monastir, TUN

**Keywords:** diabetes mellitus, diabetic neuropathies, exercise, neuralgia, pain

## Abstract

Diabetic neuropathic pain (DNP) is a common and debilitating complication of diabetes mellitus, resulting from complex metabolic, vascular, and neuroinflammatory mechanisms. Pharmacological treatments provide limited relief, highlighting the need for adjunctive therapies. This review aimed to summarize current understanding of the pathophysiology of DNP and to evaluate the efficacy and mechanisms of physical activity (PA) in its management. A narrative synthesis of recent preclinical and clinical studies investigating the impact of exercise on DNP was conducted. Studies suggested that PA may improve insulin sensitivity, reduce oxidative stress, enhance microvascular blood flow, modulate neuroinflammation, and promote nerve regeneration. Clinical evidence demonstrates that aerobic, resistance, and multimodal exercise programs reduce pain severity, improve nerve conduction, and enhance functional outcomes. PA remains a safe and effective means in addition to pharmacological treatment in DNP. Tailored supervised exercise programs are recommended to optimize benefits. High-quality studies are still needed to establish standardized exercise protocols and long-term outcomes.

## Introduction and background

Diabetes mellitus is a chronic metabolic disorder characterized by hyperglycemia resulting from defects in insulin secretion, insulin action, or both. Its global prevalence continues to rise, with the International Diabetes Federation estimating that 537 million adults were living with diabetes in 2021, a figure projected to reach 783 million by 2045 [1]. Among its chronic complications, peripheral neuropathy (PN) is one of the most common, affecting up to 50% of patients with long-standing disease [2].

A particularly disabling manifestation of PN is diabetic neuropathic pain (DNP), which occurs in approximately 13-26% of individuals with diabetes [3]. Its prevalence increases with age, particularly among type 1 diabetic patients, where it nearly doubles across older age categories, with a similar but less pronounced trend in type 2 diabetes. Women also appear to be at higher risk, showing about a 50% greater likelihood of developing painful symptoms compared with men. In contrast, no consistent association has been reported with body mass index [3]. DNP is associated with burning, stabbing, or electric shock-like sensations, often accompanied by increased sensitivity to normally non-painful stimuli (allodynia) or exaggerated pain responses to painful stimuli (hyperalgesia). Although DPN is seldom considered life-threatening, it can induce significant disabilities, daily activity restrictions, sleep disturbances, depression, and alteration of quality of life (QoL) [4].

Current pharmacological management, including anticonvulsants, antidepressants, and topical agents, provides limited symptom relief with non-negligible side effects [5]. Consequently, there is growing interest in non-pharmacological approaches that may address both symptoms and DPN pathophysiology. Among these alternatives, physical activity (PA) has emerged as a promising intervention. PA is biologically plausible in this context because it improves insulin sensitivity, endothelial function, and microvascular perfusion, processes that may reduce nerve damage and oxidative stress, thereby alleviating neuropathic pain [6,7]. While the analgesic and neuroprotective effects of PA have been demonstrated in preclinical models [8], recent clinical trials and meta-analyses suggest that structured exercise programs may also lead to meaningful reductions in pain intensity and improvements in nerve conduction in humans [9-11].

This narrative review aims to synthesize current evidence from preclinical and clinical studies to elucidate the mechanisms and therapeutic potential of PA in managing DNP. The following sections summarize the underlying mechanisms of DNP, the mechanistic effects of PA, and the available clinical evidence supporting its therapeutic role.

## Review

Methods

Given the narrative nature of this review, no quantitative synthesis, meta-analysis, or statistical pooling was performed; all evidence is summarized qualitatively using a thematic narrative approach. A focused search was conducted in PubMed, Scopus, and Google Scholar up to December 2024 using combinations of terms related to PA, exercise, diabetic neuropathy, and neuropathic pain. Priority was given to high-quality empirical studies, authoritative reviews, and key conceptual contributions. Studies were selected based on how well they addressed pathophysiological processes and treatment outcomes. Evidence was synthesized thematically to provide an integrative overview of the role of P in the management of DNP.

Pathophysiological mechanisms of DNP

While pain stands out as a primary symptom of diabetic neuropathy, the exact pathophysiological mechanisms behind it remain incompletely understood. DNP results from complex, multifactorial pathophysiological processes involving both peripheral and central nervous system perturbations. Hyperglycemia-induced metabolic disturbances, vascular insufficiency, neuroinflammation, and dysfunctional neural plasticity contribute collectively to nerve damage and pain perception abnormalities [12,13].

Microvascular Dysfunction

Microvascular dysfunction plays a pivotal role, where chronic hyperglycemia leads to thickening and fibrosis of the basal lamina in the small vessels supplying peripheral nerves. This results in nerve ischemia and subsequent degeneration, particularly affecting small sensory fibers responsible for pain and temperature sensation [14]. Endothelial dysfunction further exacerbates impaired nerve perfusion, reducing nutrient and oxygen delivery [15].

Metabolic Factors

Metabolic factors also play a significant role in DNP by increasing the flux through the polyol pathway, which consumes cofactors such as nicotinamide adenine dinucleotide phosphate. This results in a reduction of the antioxidant capacity by depleting glutathione and promoting oxidative stress via the accumulation of advanced glycation end-products [16]. Oxidative damage impairs mitochondrial function and triggers neuronal apoptosis, aggravating neuropathic damage [17].

Neuroinflammation

Besides, neuroinflammation, mediated by the activation of glial cells in the dorsal root ganglion (DRG) and spinal cord, sustains chronic pain states. In fact, microglial activation leads to the release of pro-inflammatory cytokines (e.g., TNF-α, IL-1β, IL-6), chemokines, and reactive oxygen species, fostering a pro-nociceptive environment that sensitizes peripheral and central neurons [18,19]. This inflammatory condition contributes to both peripheral and central sensitization, amplifying pain responsiveness even in the absence of peripheral stimuli [19].

Neural Plasticity Dysfunction

Neuropathic pain arises from damage or potential harm to the somatosensory system, causing maladaptive nociception. Typically, in response to a harmful stimulus, nociceptive neurons convey signals from the injury site through the DRG to the spinal cord and eventually reach the brain. These ascending inputs undergo central processing, and descending outputs travel down the spinal cord to the dorsal horn, modulating pain signals through both excitatory and inhibitory neurons. In neuropathic pain, a defining feature is the experience of pain without a stimulus, thought to result from nerve damage inducing peripheral nerve sensitization (hyperexcitability) and/or central sensitization [20]. In addition, neurogenesis impairment in brain regions such as the hippocampus has been associated with chronic neuropathic pain and cognitive disturbances in diabetic patients [21].

Furthermore, several risk factors, such as deteriorating glucose tolerance, advanced age, longer duration of diabetes, and habits like alcohol consumption and cigarette smoking, are linked to DNP. Overall, DNP emerges from a synergistic interaction of vascular, metabolic, inflammatory, and neuroplastic changes that disrupt normal nociceptive processing, resulting in chronic and functionally impairing pain.

Analgesic mechanisms and effects of PA on DNP

While numerous treatments aim to relieve DNP, an increasingly recognized and promising strategy involves incorporating PA into the management of this complication. PA is characterized by ongoing bodily movements involving the contraction of skeletal muscles, leading to energy expenditure in daily life. It encompasses a wide range of actions, including exercises, movements performed during daily tasks, and structured sports or voluntary leisure activities. The principal objective of PA, from a rehabilitation perspective, is to enhance overall physical function, promote mobility, and contribute to the restoration of health and well-being. The specific recommendations for PA may vary based on an individual's medical condition, rehabilitation aims, and overall health status [22].

PA exerts multifaceted beneficial effects on DNP through metabolic, vascular, anti-inflammatory, neuroplastic, and analgesic pathways. These mechanisms collectively address key pathophysiological processes underlying DNP and contribute to symptom amelioration and functional improvement [22,23].

Metabolic Regulation

First, PA is known to enhance glycemic regulation by improving insulin sensitivity, mitigating hyperglycemia-induced nerve damage, and preventing the progression of diabetic neuropathy [24]. Enhanced glucose uptake by skeletal muscles reduces the activation of the polyol pathway and formation of advanced glycation end-products, thereby decreasing oxidative stress and preserving mitochondrial function in peripheral nerves. Thus, central neuronal injury patterns and maladaptive pain signaling may be controlled, resulting in an improvement of DNP [25].

Microvascular Dysfunction Improvement

Furthermore, exercise stimulates blood flow and endothelial nitric oxide production, fostering better circulation throughout the organism, including peripheral nerves. This enhanced blood flow ensures an adequate supply of oxygen and nutrients to nerve tissues, supporting their function and diminishing the risk of ischemia-related pain. As a result, it provides neuroprotective benefits by promoting the growth and survival of nerve cells and participating in nerve regeneration [26,27].

Anti-inflammatory Action

It has been shown that PA has an important impact on the inflammatory component of DNP [28]. Hence, it modulates systemic and local inflammation by decreasing pro-inflammatory cytokines (e.g., TNF-α, IL-1β, IL-6) and increasing anti-inflammatory mediators such as interleukin-10 and transforming growth factor-beta [28]. This shift reduces neuroinflammation and glial activation in the DRG and spinal cord, thereby attenuating peripheral and central sensitization associated with neuropathic pain [29].

Neuroplasticity and Nerve Regeneration

PA exerts a modulatory effect on pain-related neuroplasticity by influencing both peripheral and central mechanisms of nociceptive processing. At the peripheral level, PA reduces pro-inflammatory cytokines, oxidative stress, and metabolic dysfunction, thereby limiting maladaptive signaling from injured nerves [30]. Centrally, regular PA enhances inhibitory neurotransmission, promotes the release of endogenous opioids, and restores the balance between excitatory and inhibitory circuits within the spinal cord and brain regions implicated in pain processing. PA has also been shown to induce neurotrophic factors such as brain-derived neurotrophic factor (BDNF), which support synaptic remodeling and adaptive plasticity, counteracting the sensitization processes that underlie chronic neuropathic pain [31,32].

PA also promotes neurotrophic factor expression, including BDNF and nerve growth factor, which support neuronal survival and axonal regeneration [32,33]. These neuroplastic changes enhance peripheral nerve repair and may counteract the maladaptive central changes involved in chronic pain processing [34].

Endogenous Analgesia

One of the most important analgesic effects is the endorphin-mediated pain modulation. PA induces the release of endogenous opioids such as endorphins and enkephalins, which bind to opioid receptors in the central nervous system to inhibit pain transmission [35]. Additionally, exercise elevates monoamine neurotransmitters like serotonin and norepinephrine, further modulating pain perception and improving mental health status and overall well-being [36].

**Figure 1:**
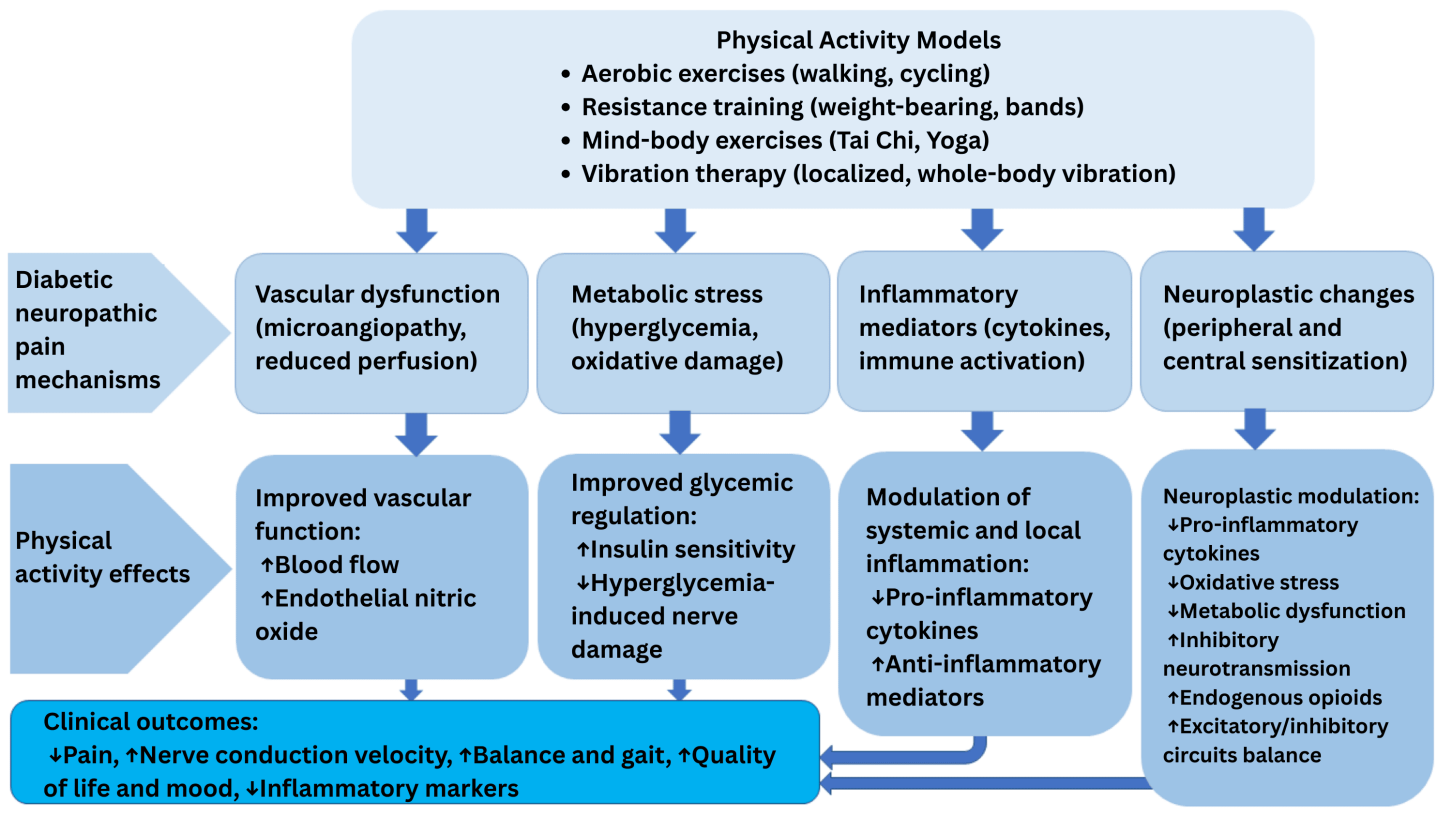
PA effects on DNP PA: physical activity, DNP: diabetic neuropathic pain Image credit: Dr. Amr Chaabeni and Dr. Anis Jellad

In conclusion, PA addresses multiple interconnected mechanisms contributing to DNP pathogenesis (Figure 1), rendering it a promising adjunctive intervention to pharmacological treatments. These pathophysiological insights are substantiated by both preclinical and clinical studies demonstrating symptom alleviation and functional improvements following exercise interventions in diabetic patients with neuropathic pain [37].

Evidence from clinical and preclinical studies

The efficacy of PA in alleviating DNP has been explored in both preclinical models and human clinical studies. This evidence reinforces the mechanistic rationale outlined above and provides insight into optimal exercise modalities and outcomes.

Preclinical Studies

Animal models of diabetic neuropathy have been instrumental in elucidating the physiological effects of exercise on neuropathic pain. For example, studies in diabetic rodents demonstrate that forced exercise reduces pain behaviors, improves nerve conduction velocities, and enhances peripheral nerve regeneration [14,38]. These effects are mediated through modulation of voltage-activated calcium channels, reduction of oxidative stress, and suppression of neuroinflammatory markers [25,33]. Exercise-induced upregulation of neurotrophic factors such as BDNF has also been reported, further supporting nerve repair and functional recovery [34].

Clinical Evidence

Recent systematic reviews, meta-analyses, and randomized controlled trials (RCTs) have investigated the effects of various PA interventions on DNP symptoms and associated functional outcomes. A scoping review by Pebrianti et al. synthesized evidence from multiple intervention types, including aerobic exercise, resistance training, vibrotherapy (plantar and whole-body vibration), and Tai Chi [7]. The review concluded that most exercise modalities significantly reduced pain scores and improved balance and QoL in patients with diabetic neuropathy, with aerobic and resistance exercises being most commonly studied and effective [7].

Furthermore, an umbrella review published in 2025 consolidated findings from 13 systematic reviews and meta-analyses. It confirmed that exercise interventions generally improve neuropathic pain intensity, nerve conduction parameters, glycemic control, balance, and physical function in patients with DPN. However, effects on QoL and ulcer risk were more variable, highlighting the need for individualized exercise protocols and longer follow-up durations [11].

More specifically, a recent meta-analysis found that moderate-intensity exercise performed for approximately eight weeks yielded significant improvements in nerve conduction velocities, especially in the lower limbs [39]. The analysis suggested that patients with shorter diabetes duration (≤5 years) derived greater benefit, emphasizing the importance of implementing intervention early [39].

In addition to these reviews, recent RCTs have explored novel and combined exercise programs [9,17,18]. It appears that a multisystem exercise protocol incorporating balance, proprioception, muscle strengthening, and reaction time training demonstrated superior improvements in pain severity, functional mobility, and balance compared to conventional aerobic exercise alone. These findings emphasize the potential advantages of comprehensive, multimodal exercise approaches tailored to individual patient needs [9].

These converging lines of evidence support the role of PA as a beneficial adjunct in the management of DNP, highlighting its multifactorial benefits and informing clinical practice.

Clinical implications and recommendations

The accumulating evidence highlights PA as a safe, effective, and multifaceted adjunct in the management of DNP. Incorporating tailored exercise programs into routine clinical care can yield meaningful reductions in pain severity, improve nerve function, and enhance overall QoL for diabetic patients [7,9].

Exercise Prescription and Safety Considerations

Exercise prescriptions for DNP should be individualized, reflecting variability in disease severity, functional capacity, and comorbidities. Aerobic and resistance training remain the cornerstone, with evidence showing improvements in glycemic control, peripheral nerve function, and pain reduction [24,27]. In contrast, multimodal programs incorporating balance and proprioceptive training are particularly beneficial in those with advanced neuropathy or gait instability [9]. A pragmatic target is 150 minutes of moderate-intensity aerobic activity per week, divided into manageable bouts, with resistance training introduced two to three times weekly, progressing cautiously under supervision where available [40]. Low-impact options such as Tai Chi, seated exercises, or aquatic training can broaden accessibility for patients with mobility limitations or heightened pain [7]. Safety considerations are paramount: clinicians should conduct pre-exercise screening for cardiovascular risk, musculoskeletal restrictions, and foot integrity; emphasize protective footwear; and teach patients daily foot inspections to minimize ulceration or injury risk [41]. Progressive overload should be gradual, with adaptation based on patient tolerance and symptom monitoring. Community health workers or trained exercise facilitators can substitute for specialist supervision in resource-limited settings. Embedding patient education on pacing, self-monitoring, and injury prevention enhances adherence and supports safe long-term engagement in PA [42].

Integration of PA With Pharmacological Treatments and Individual Factors

Integration of PA with pharmacological treatments offers a pragmatic, complementary strategy for DNP management. Rather than replacing pharmacotherapy, structured PA enhances its efficacy. Evidence suggests additive or even synergistic effects on pain relief, glycemic control, and functional outcomes, thereby allowing potential reductions in drug dosage and mitigating adverse effects [43]. Importantly, PA also confers broad systemic benefits by lowering cardiovascular risk, improving insulin sensitivity, and supporting weight management, factors particularly relevant to patients with diabetes who frequently present with multimorbidity [43]. Clinicians in primary care should frame PA as a routine “co-therapy,” recommending sustainable activities such as walking, resistance training, or low-impact modalities alongside first-line agents like duloxetine or pregabalin. Patient education should emphasize that exercise adherence amplifies the benefits of pharmacological treatment and may improve long-term outcomes while reducing reliance on medication.

Individual characteristics modulate the response to PA. Younger patients, particularly children and adolescents with type 1 diabetes, tend to experience greater improvements in BMI, glycemic markers, and physical fitness following long-term exercise programs due to higher metabolic adaptability and fewer comorbidities [44]. Older adults with type 2 diabetes and longer disease duration may exhibit slower or attenuated responses, influenced by sarcopenia and polypharmacy. Gender differences have also been observed: women show better adherence to low-intensity aerobic or balance programs, whereas men demonstrate greater gains in muscle mass and insulin sensitivity from resistance training [45]. Baseline BMI further modulates response, with overweight or obese individuals benefiting more from combined aerobic-strength interventions due to effects on body composition and vascular function. At the same time, normal-weight patients achieve pronounced glycemic improvements from strength training alone [46]. Coexisting conditions, such as cardiovascular or peripheral arterial disease, may necessitate individualized exercise prescriptions emphasizing aerobic capacity and safety monitoring [46]. Importantly, the combination of aerobic and strength training has been identified as the most effective approach for reducing HbA1c and improving other metabolic parameters, surpassing interventions that use only one type of exercise [47]. Also, participation in regular physical exercise, maintaining good glycemic control, and consistent adherence to antidiabetic therapy are all positively associated with better clinical outcomes [48].

Overall, integrating tailored PA regimens with pharmacological therapy optimizes outcomes across diverse patient profiles, enhancing both metabolic control and neuropathic pain management while ensuring a safer, more sustainable model of care.

Integration of PA in Rehabilitation Programs and Future Directions

Integrating PA into structured rehabilitation programs represents a promising approach for DNP, complementing conventional medical care [49]. Evidence supports the incorporation of aerobic training, Tai Chi, and mindfulness-based interventions, which have shown benefits for pain reduction and functional improvement, while multimodal rehabilitation combining PA with physiotherapy, balneotherapy, and natural therapeutic factors may further enhance gait and quality of life [50]. Such programs should be designed as periodic, comprehensive interventions, adaptable to patient needs, resources, and comorbidities, with emphasis on progressive training and ongoing patient education. Looking ahead, large-scale RCTs are needed to refine optimal exercise parameters, including modality, intensity, frequency, and duration, and to assess their sustained effects on neuropathic pain, nerve regeneration, and psychosocial outcomes. Future directions should also prioritize the integration of digital health solutions, such as wearable activity trackers, mobile applications, and tele-rehabilitation platforms, to improve accessibility, monitoring, and adherence, particularly in underserved or rural settings. Embedding PA within rehabilitation pathways not only addresses neuropathic symptoms but also contributes to holistic diabetes management, thereby supporting long-term functional independence and quality of life [17].

## Conclusions

DNP is a multifactorial condition driven by metabolic dysregulation, microvascular compromise, neuroinflammation, and maladaptive neuroplasticity. PA offers a valuable adjunctive strategy, addressing these mechanisms while improving pain, nerve function, glycemic control, cardiovascular health, and overall quality of life. Individualized, supervised programs that combine aerobic, resistance, and balance training remain the most effective and safest approach. Moreover, engagement in structured physical exercise, optimal glycemic control, and adherence to prescribed antidiabetic therapies have been consistently associated with improved clinical and metabolic outcomes in patients with DNP. However, future research must prioritize the development of standardized, evidence-based exercise protocols, as current heterogeneity in program design limits comparability and translation into practice. Large-scale, multicenter RCTs are needed to establish.
